# Trunk, head and pelvis interactions in healthy children when performing seated daily arm tasks

**DOI:** 10.1007/s00221-018-5279-2

**Published:** 2018-05-08

**Authors:** L. H. C. Peeters, I. Kingma, G. S. Faber, J. H. van Dieën, I. J. M. de Groot

**Affiliations:** 1 Department of Rehabilitation, Donders Centre for Neuroscience, Radboud University Medical Center, 9101, 6500 HB Nijmegen, The Netherlands; 2 Department of Human Movement Sciences, Faculty of Behavioral and Movement Sciences, Vrije Universiteit Amsterdam, Amsterdam Movement Science, Amsterdam, The Netherlands

**Keywords:** Trunk, Head, Kinematics, Coordination, Sitting, Reaching

## Abstract

Development of trunk and head supportive devices for children with neuromuscular disorders requires detailed information about pelvis, trunk and head movement in interaction with upper extremity movement, as these are crucial for daily activities when seated in a wheelchair. Twenty-five healthy subjects (6–20 years old) were included to obtain insight in the physiological interactions between these segments and to assess maturation effects. Subjects performed a maximum range of trunk and head movement tasks and several daily tasks, including forward and lateral reaching. Movements of the arms, head, pelvis, and sub-sections of the trunk were recorded with an optical motion capture system. The range of motion of each segment was calculated. Contributions of individual trunk segments to the range of trunk motion varied with movement direction and therefore with the task performed. Movement of pelvis and all trunk segments in the sagittal plane increased significantly with reaching height, distance and object weight when reaching forward and lateral. Trunk movement in reaching decreased with age. Head movement was opposite to trunk movement in the sagittal (> 50% of the subjects) and transverse planes (> 75% of the subjects) and was variable in the frontal plane in most tasks. Both trunk and head movement onsets were earlier compared to arm movement onset. These results provide insight in the role of the upper body in arm tasks in young subjects and can be used for the design of trunk and head supportive devices for children with neuromuscular disorders.

## Background

Children with neuromuscular disorders (NMD) suffer from progressive muscle weakness. Generally, they first lose the ability to walk, followed by a decrease in trunk and arm function. Some children, e.g., with spinal muscular atrophy type I or II, may never have the ability to walk, while patients with Duchenne muscular dystrophy lose the ability to walk around the age of 12 years (van den Bergen et al. 2014). When seated in a wheelchair, the autonomy and level of independence are highly dependent on arm function (Natterlund and Ahlstrom 2001). Patients report that eating and drinking, reaching for objects, writing and personal hygiene are most problematic in daily life and therefore assisting performance of these tasks with supportive devices is of key importance (Janssen et al. 2014).

In addition to control of upper extremity movement, trunk and head control are necessary in accomplishing daily tasks. The interaction between trunk and arm movements is likely most pronounced when reaching to objects beyond arm length distance (Schneiberg et al. 2002; Sveistrup et al. 2008). However, in healthy children, trunk movement is also seen when performing tasks within arm length distance (Schneiberg et al. 2002; Coluccini et al. 2007). Furthermore, trunk motions are often needed to maintain postural stability during daily tasks (Flatters et al. 2014). In healthy children and adults, the head generally shows a countermovement relative to the trunk resulting in a constant head orientation in space (Sveistrup et al. 2008). Head movement is also important for visual control of task performance. Maturation affects the interactions between arm, trunk and head movements in children. Interactions in younger children are more variable than in older children (Sveistrup et al. 2008).

When developing supportive devices for patients with NMD, trunk and head as well as arm movement should be taken into account. Therefore, detailed information is needed about pelvis, trunk and head movement in coordination with arm movements, both in healthy children and in children with NMD. However, literature on these segmental interactions is scarce (Peeters et al. 2018). In our study, healthy children in the same age range as children with NMD were included to obtain insight in the interaction between upper body segmental movements, prior to studying this in children with NMD.

While there is some knowledge on the interactions of the upper body in healthy children, the trunk is mostly regarded as one rigid segment. The movement of the thorax is often measured, with respect to the pelvis or the world, and is seen as representative for the overall trunk movement. However, the trunk has great flexibility and can probably not be seen as a rigid segment for development of dynamic supportive devices. Clearly, for the development of supportive devices or spinal orthoses, it is important to have insight in the movement of the trunk in more detail than as a single segment. This information could result in requirements concerning selection which trunk segment movements should be allowed to move or be supported when performing daily activities.

Therefore, our aim was to obtain more insight in the interaction of trunk, head and arm movements in healthy children with a specific focus on the segmental nature of the trunk.

## Methods

### Participants

Twenty-five healthy children and young adults (13 males, 6–20 years) participated in this study. The subjects were evenly distributed over the age range. None of the participants had a history of disorders affecting movement of the upper body. In addition, they had no scoliosis and no pain in arm(s), trunk, neck or head at the time of participation.

Participants were recruited from local primary schools, high schools and university. Prior to participation, informed consent was given by participants when over 12 years old, and by the children’s parents or guardians for all participants younger than 18 years old. The study was approved by the medical ethics committee Arnhem-Nijmegen (NL53143.091.15) and all data were handled according to the guidelines of good clinical practice.

### Experimental setup

All subjects were seated on a height adjustable chair with a multi-celled air cushion (Starlock, Star Cushion Products, Freeburg, IL, USA), without back- or armrests. Before the measurement, the cushion was formed to each individual shape by releasing air to provide some additional sitting stability and comfort. The sitting height was adjusted so that the knees were flexed 90° and both feet were flat on the ground.

**Fig. 1:**
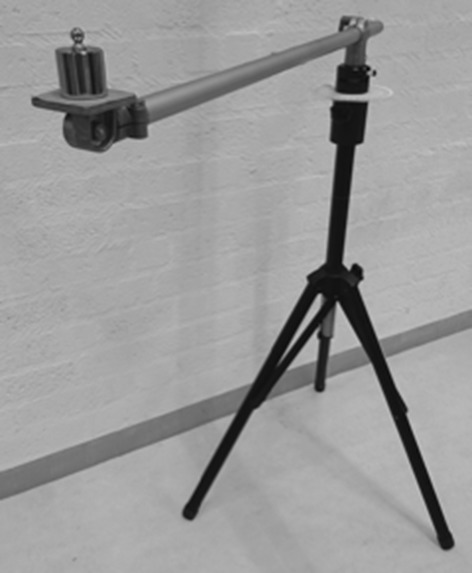
Reference frame with 500 g weight used for performing the reaching tasks

First, subjects were asked to perform a maximum flexion movement of their trunk from a seated position, immediately followed by a maximum extension movement of their trunk (keeping both feet on the ground). They were instructed to move from the upright position to the maximum position at a slow pace (3 s) and repeated this flexion–extension movement three times. The same was done for maximum axial rotation and lateral bending. The arms were crossed at the chest when performing the flexion–extension and rotation task, and were rested on the upper legs when performing the lateral bending task. No instructions were given regarding pelvis or hip movement. Subsequently, movements were repeated for the head. Here the instruction was to keep the rest of the body as quiet as possible and only move the head. Thereafter, a series of tasks was performed with the dominant hand at a self-selected speed. No instructions were given for the other hand. Several reaching (and placing) tasks were performed: reaching forward, sideways and contra-lateral at a 45° angle in the transverse plane. The subjects were asked to touch a reference frame positioned at the desired position, or to place a weight on the reference frame (Fig. 1). Reaching distance, height and object weight were varied, resulting in the following combinations for forward and lateral reaching: nearby-shoulder height-0 g (“N-S-0”), nearby-shoulder height-500 g (“N-S-500”), far-shoulder height-0 g (“F-S-0”), nearby-eye height-0 g (“N-E-0”), nearby-eye height-500 g (“N-E-500”), far-eye height-0 g (“F-E-0”). Contra-lateral reaching was only performed nearby-shoulder height-0 g and nearby-shoulder height-500 g. Nearby was defined as 100% arm length, far as 133% arm length. Arm length was defined as the distance from mid-acromion to mid-hand. Furthermore, subjects were asked to perform four daily tasks: displace a porcelain plate from left to right on a table with both hands (“Plate”), bring a cup of 200 g to the mouth (“Drink”), trace a path with a pencil (“Draw”) and place a finger on a number diagram while holding the diagram with the other hand (“Dexterity”). The drink, draw and dexterity task were based on the instructions of the performance of the upper limb (Mayhew et al. 2013). No instructions were given on how to perform the tasks.

### Data acquisition

**Fig. 2:**
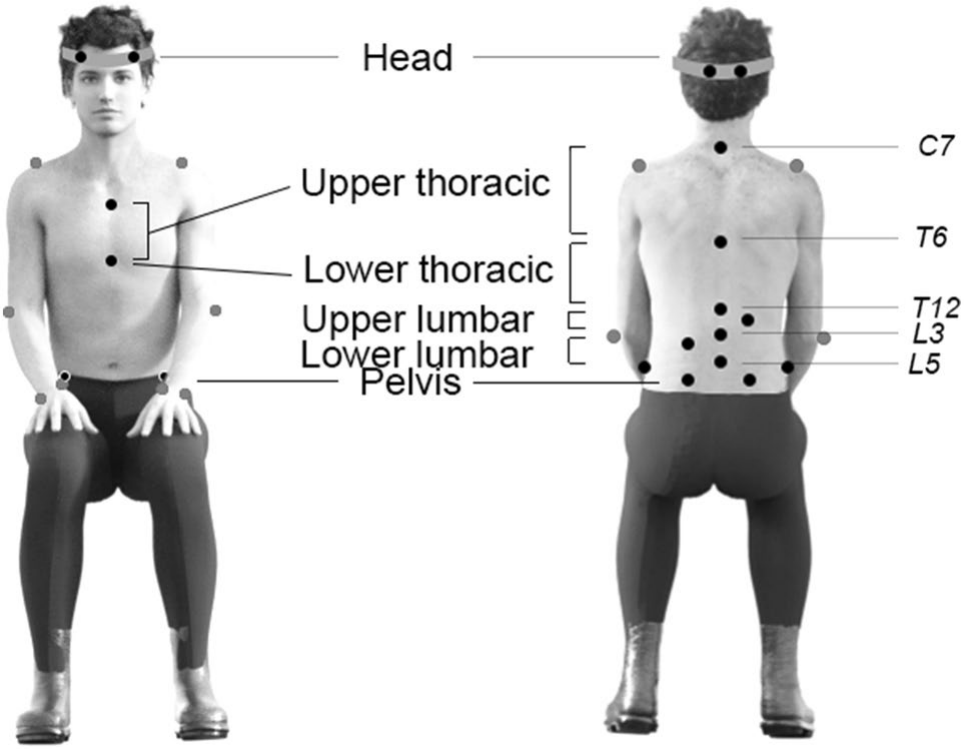
Illustration of marker placement

Marker positions were recorded at 100 samples/s using an optical motion capture system (Vicon, Oxford, UK). Twenty-five reflective markers were placed on the skin to define the position of the head, trunk, pelvis and both arms (Fig. 2). The trunk was divided into four segments (upper thoracic, lower thoracic, upper lumbar and lower lumbar) to obtain a detailed representation of trunk movement (Schinkel-Ivy and Drake 2015). Markers on the head, pelvis and arms were placed according to the Vicon Plugin-Gait model. For 15 subjects, two additional markers were placed on both sides on the iliac crest, as we noticed that the anterior superior iliac spine markers often became invisible when flexing the trunk or moving the arms. The upper thoracic segment was defined by markers on spinous processes of the 7th cervical vertebrae (C7), spinous processes of the 6th thoracic vertebrae (T6), jugular notch and xiphoid process of the sternum. The lower thoracic segment was defined by markers on T6, spinous processes of the 12th thoracic vertebrae (T12) and the xiphoid process. The upper lumbar segment was defined by markers on T12, spinous processes of the 3rd lumbar vertebrae (L3) and a laterally placed marker at the level of the 1st/2nd lumbar vertebrae. The lower lumbar segment was defined by markers on L3, spinous processes of the 5th lumbar vertebrae (L5) and a laterally placed marker at the level of the 4th lumbar vertebrae.

### Data analysis

Data were filtered with a bi-directional 4th order Butterworth low-pass filter (cutoff frequency of 6 Hz). A biomechanical model was used to calculate the movements of the body segments (Kingma et al. 1996). Joint coordinate systems were based on the ISB-guidelines (Wu et al. 2002, 2005). The longitudinal axis was created first for the trunk segments and the following kinematic variables were extracted using Euler decomposition in the following order:

- Pelvis angle: angle of the pelvis relative to the global coordinate system (anterior/posterior tilt–lateral tilt–axial rotation)
- Individual trunk segment angles: angle of a trunk segment relative to the more caudal segment (flexion/extension–lateral bending–axial rotation).
- Neck angle: angle of the head relative to the upper thoracic segment (flexion/extension–lateral bending–axial rotation)

Flexion, lateral bending to the right and rotation to the right were defined as positive. Movements of the different trunk segments were named after the more cranial segment (e.g., upper thoracic angle represents the orientation of the upper thoracic segment relative to the lower thoracic segment). ‘Total trunk movement’ is used for the summation of all trunk segments.

Pelvis, trunk and neck angles during a recording while sitting quietly [i.e., sitting upright with both hands on the legs (see Fig. 2)] were used to zero angles in the movement trials. This was done by post-multiplying the orientation matrix of all segments with the inverse of the orientation matrix while sitting quietly. All kinematics for the two left-hand dominant subjects were transformed to match the kinematics for the right-hand dominant subjects.

To determine maximum ranges of trunk motion, the trial in which the summed angle of all trunk segments and pelvis was maximal in the requested movement plane was selected. Similarly, the trial with the maximum range of neck motion was selected.

For all reaching tasks, the instant of task execution that was used for analyses of segment angles was defined as the first instant where the wrist velocity reached zero after the maximum wrist velocity. For the drink task, this instant was at the point where the hand was the closest to the mouth (i.e., peak of the wrist movement path) and for the plate task, this was where the hands grabbed the plate on the left side (i.e., peak of right wrist movement path). For all of these tasks, the start was identified as the instant where the velocity of the wrist exceeded 5% of its peak velocity. All instants were selected by a computer algorithm and afterwards visually confirmed. For the drawing and dexterity tasks, the instant at task execution was midway between start and end. Start and end were defined manually with the use of video and kinematics recordings, since rendering automatic detection was unfeasible due to low wrist velocity. The ROM was defined as the segment angles at the instant of task execution, subtracted by the segment angles at the start position of the same task. Kinematics of the arms are not reported in this article.

Head movements relative to the upper thoracic segment were categorized in three different strategies: no relative movement between head and trunk, relative movement of the head in the same direction as the trunk, or relative movement of the head in opposite direction to the trunk. The range where the head movement was defined none, was in between plus or minus two times the standard deviation obtained from the head movement during the quiet sitting task. The maximum standard deviation of all participants, in each direction was used for this. For each subject and trial, the head strategy was determined and the percentage of subjects using each strategy was calculated.

Movement onsets of the head and trunk were defined relative to hand movement onset for the reaching tasks, based on 5% of their respective peak velocities. The midpoint between the front head markers was used to determine movement onset of the head, the marker at the jugular notch of the sternum for the movement onset of the trunk and the midpoint between the wrist markers for the movement onset of the hand.

All analyses were performed using Matlab R2014b (Math Works, USA) software.

### Statistics

Statistical analyses were performed in SPSS 22.0. Non-parametric tests were used, since most of the data was not normally distributed. One-way ANOVA with a Bonferroni-corrected post hoc test, was used to assess differences in ROM between segments when performing maximum trunk movements and when performing daily tasks. Wilcoxon signed rank tests were used to evaluate differences between rotations to the left and right for both trunk and head. A Friedman test, followed by a Wilcoxon signed rank test in case of a significant effect, was used to evaluate the effect of reaching height, distance and object weight on the ROM.

Linear regression analysis was performed to evaluate the correlation between subject age and trunk movement and the effect of age on trunk movement, when performing forward and lateral reaching tasks.

A one-sample Wilcoxon signed rank test was used to evaluate whether the trunk and head movement onset differs from zero (i.e., arm movement onset). The difference between trunk and head movement onsets was evaluated with a two-sample Wilcoxon signed rank test.

The statistical level was set at *α* = 0.05 for all analysis.

## Results

Each movement task was successfully completed by all subjects, with the exception of reaching laterally, 1.3 times arm length at eye level. In this task, none of the subjects was able to reach the target and the target was repositioned to their maximum reach distance. Out of 25 subjects, subject data for one subject (12 of 128 kinematic outcomes), for two subjects (13 of 128 kinematic outcomes), and for three subjects (7 of 128 kinematic outcomes) were excluded due to missing marker data. Kinematic outcomes consist of all segments and tasks.

### Maximum range of motion tasks

**Fig. 3:**
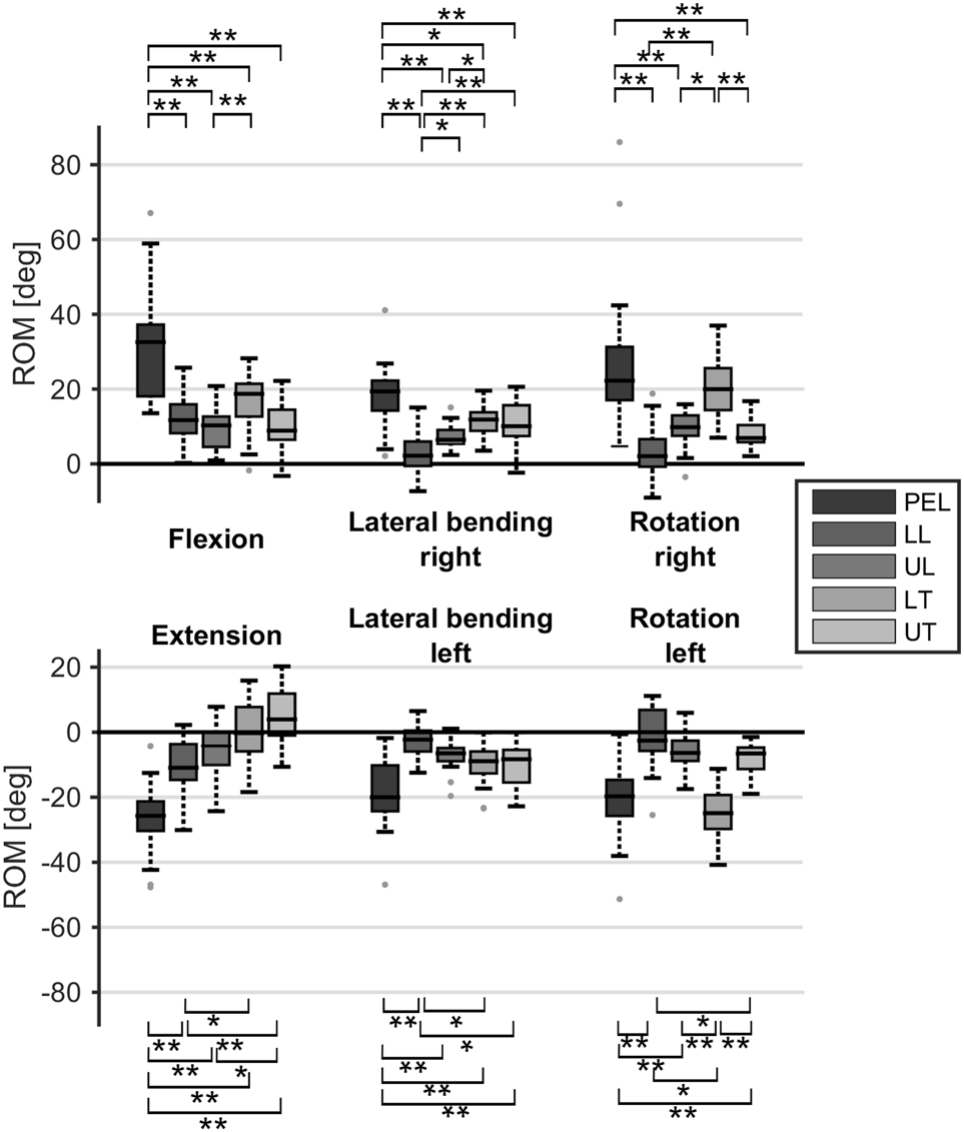
Range of motion (ROM) for pelvis and various trunk segments in the frontal, sagittal and transversal plane, when performing a maximum trunk flexion, extension, lateral bending or axial rotation task, respectively. Boxes represent 25th, 50th and 75th percentile, whiskers minimum and maximum of non-outlier values, and dots indicate outliers (greater than 1.5 times the interquartile range). *p < 0.05, **p < 0.01

The maximum pelvis and trunk ROM when performing maximum trunk movement tasks are shown in Fig. 3. In all movement directions, except for the trunk axial rotation task, the pelvis had a significantly larger contribution than all trunk segments (*p* < 0.05). The pelvis and the lower thoracic segment had the largest contribution [i.e., significantly different from the other trunk segments (*p* < 0.05)] in the axial rotation task, but were not significantly different from each other. The thoracic segments contributed more in the lateral trunk movement, compared to the lumbar segments. This difference was significant when comparing the lower lumbar segment with both thoracic segments (both *p* < 0.05). For the trunk flexion task, the contribution was distributed uniformly over all trunk segments. However, when extending the trunk, the contribution decreased from caudal to cranial segments, and the difference between the two thoracic segments and the lower lumbar segment was significant (both *p* < 0.005). The interquartile ranges for both thoracic trunk segments crossed zero, indicating that some participants showed thoracic flexion instead of extension when performing a maximum trunk extension task.

There was no significant difference between left and right total range of motion, both for lateral bending (*p* = 0.135) and axial rotation (*p* = 0.545). There was a significant difference between flexion and extension (*p* < 0.001).

**Fig. 4:**
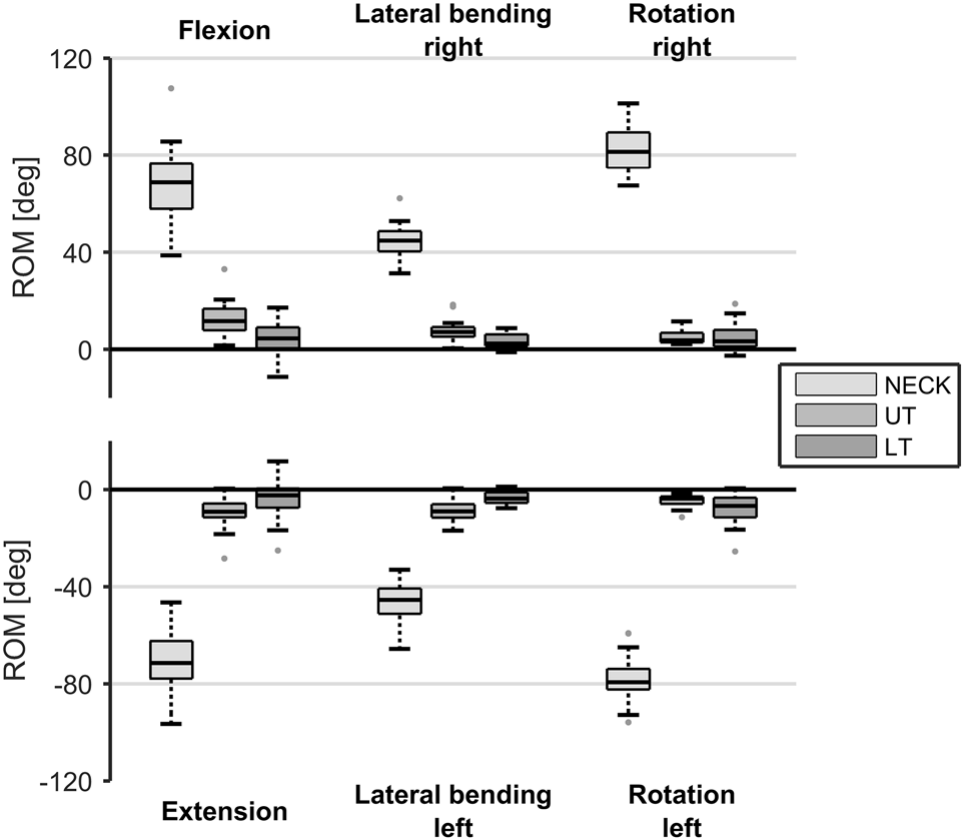
Range of motion (ROM) for neck and upper thoracic (UT) and lower thoracic (LT) trunk segments in the frontal, sagittal and transversal plane, when performing a maximum head flexion, extension, lateral bending or axial rotation task, respectively. Boxes represent 25th, 50th and 75th percentile, whiskers minimum and maximum of non-outlier values, and dots indicate outliers (greater than 1.5 times the interquartile range)

The median and interquartile ranges for maximum neck ROM are shown in Fig. 4. Notable is that also upper thoracic movement (median of 11.6°) was seen when performing the head movements. There was no significant difference between left and right lateral bending (*p* = 0.281) and axial rotation (*p* = 0.386), and flexion–extension (*p* = 0.463).

### Trunk movement in reaching and ADL

**Fig. 5:**
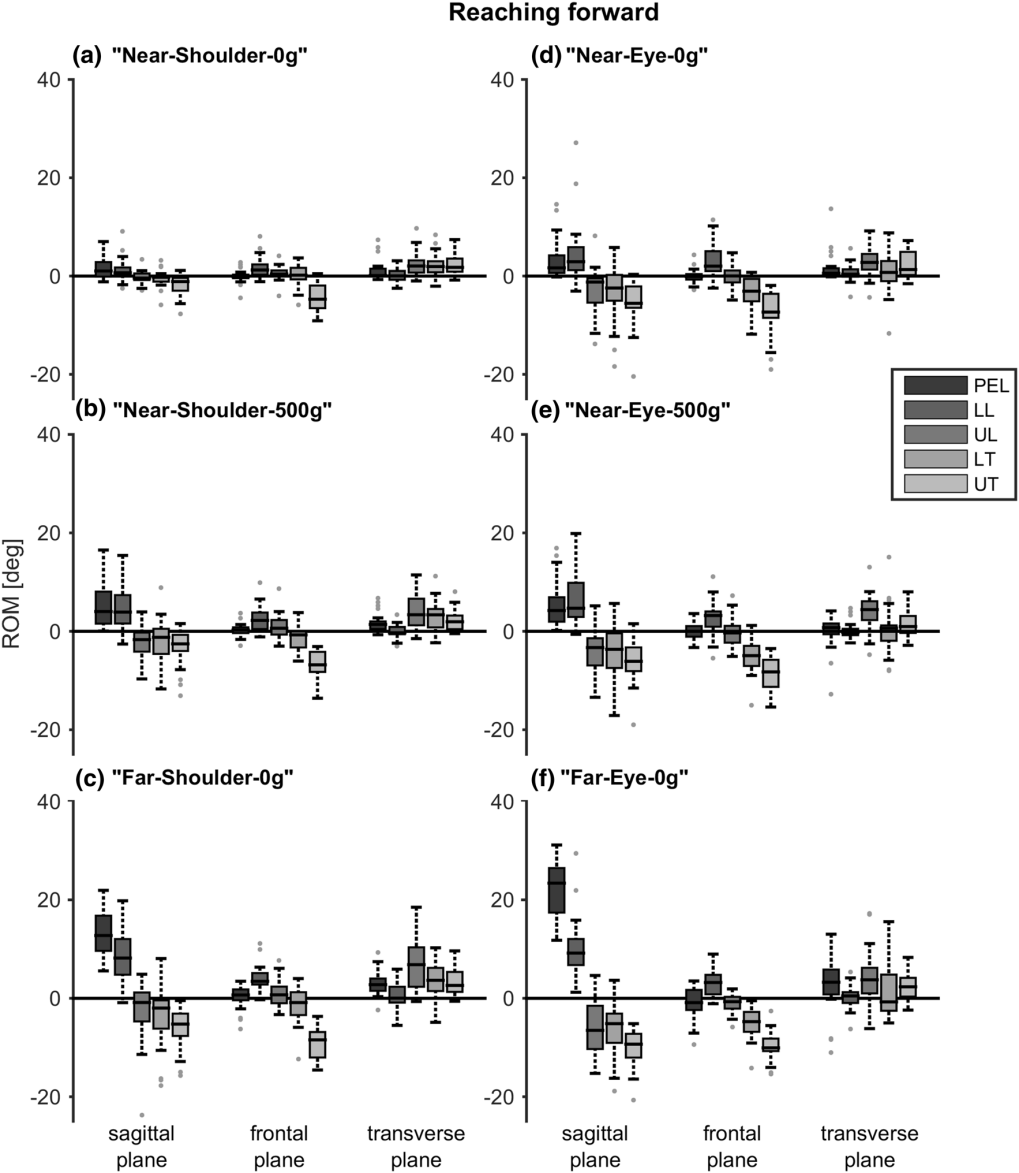
Range of motion (ROM) when reaching forward at different reaching heights, distances and object weights. Positive values indicate, respectively, flexion, lateral bending to the right and rotation to the right. Boxes represent 25th, 50th and 75th percentile, whiskers minimum and maximum of non-outlier values, and dots indicate outliers (greater than 1.5 times the interquartile range). *PEL* pelvis, *LL* lower lumbar segment, *UL* upper lumbar segment, *LT* lower thoracic segment, *UT* upper thoracic segment

When reaching forward to a target, trunk ROM in the sagittal plane increased with reaching height, distance and object weight (Fig. 5). This increase was significant for almost all segments and with all reaching conditions (Table 1). The more caudal segments (pelvis and lower lumbar segment) showed a flexion movement when reaching forward, while the more cranial segments (upper lumbar and both thoracic segments) showed an extension movement. Lateral bending significantly increased for both thoracic segments and for some reaching conditions in the lumbar segments with all reaching conditions; however, this was inconsistent between the reaching conditions (Table 1). There was no consistent, significant increase in axial rotation ROM between the reaching conditions and segments; however, quite some trunk axial rotation could be seen in all reaching tasks.

**Table 1:** *p* values for the effects of reaching height, distance and object weight on segment range of motion, when reaching forward

Pair	Reaching height			Reaching distance		Object weight	
	N-S-0/N-E-0	N-S-500/N-E-500	F-S-0/F-E-0	N-S-0/F-S-0	N-E-0/F-E-0	N-S-0/N-S-500	N-E-0/N-E-500
Segment							
Sagittal plane							
Pelvis	**0.040**	0.961	**0.006**	< **0.001**	< **0.001**	< **0.001**	< **0.001**
Lower lumbar	**0.003**	**0.010**	0.140	< **0.001**	**0.001**	< **0.001**	**0.002**
Upper lumbar	**0.009**	**0.002**	**0.014**	0.097	**0.025**	**0.016**	0.122
Lower thoracic	**0.009**	**0.002**	**0.037**	**0.006**	**0.005**	0.150	0.201
Upper thoracic	< **0.001**	**0.001**	< **0.001**	< **0.001**	< **0.001**	**0.016**	0.183
Frontal plane							
Pelvis	0.882	0.527	0.073	0.29	0.394	0.128	0.249
Lower lumbar	**0.048**	0.277	0.223	< **0.001**	0.378	**0.028**	0.592
Upper lumbar	0.078	**0.004**	**0.001**	0.689	**0.028**	0.600	0.657
Lower thoracic	< **0.001**	< **0.001**	< **0.001**	**0.035**	**0.002**	**0.009**	**0.001**
Upper thoracic	**0.001**	**0.005**	0.104	< **0.001**	**0.008**	< **0.001**	0.088
Transverse plane							
Pelvis	0.200	0.506	0.605	< **0.001**	0.144	**0.045**	0.445
Lower lumbar	0.061	0.236	0.884	0.447	0.627	0.397	0.338
Upper lumbar	0.495	0.861	**0.021**	< **0.001**	0.353	**0.020**	0.300
Lower thoracic	0.158	**0.002**	**0.004**	0.880	0.946	**0.042**	0.737
Upper thoracic	0.563	0.065	**0.002**	< **0.001**	0.264	0.619	0.581

Bold values indicate significant differences between reaching conditions (post hoc Wilcoxon test)

Abbreviations in reaching tasks: *N* near target, *F* far target, *S* shoulder height, *E* eye height, *0* 0 g object weight, *500* 500 g object weight

**Fig. 6:**
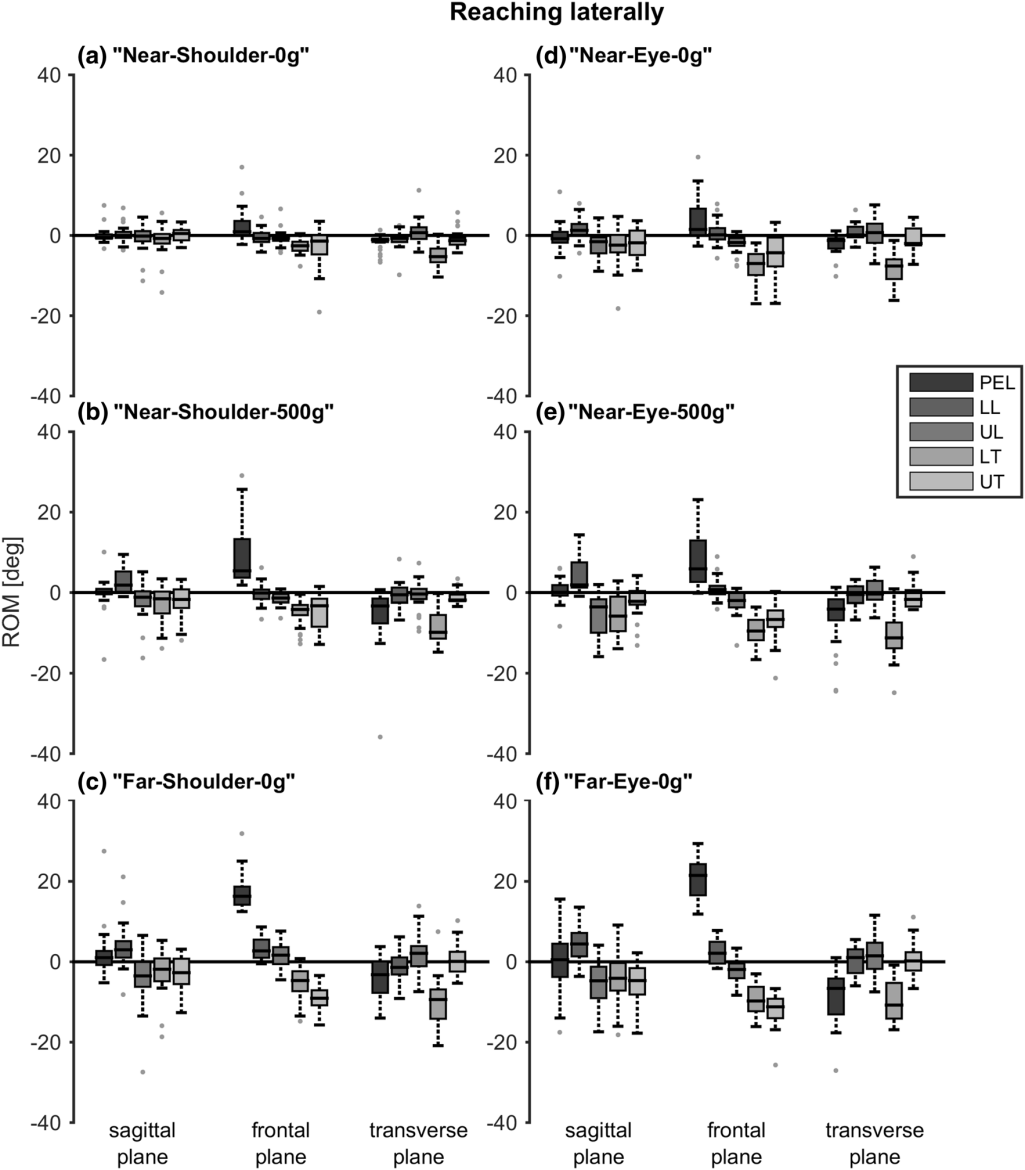
Range of motion (ROM) when reaching laterally at different reaching heights, distances and object weights. Positive values indicate, respectively, flexion, lateral bending to the right and rotation to the right. Boxes represent 25th, 50th and 75th percentile, whiskers minimum and maximum of non-outlier values, and dots indicate outliers (greater than 1.5 times the interquartile range). *PEL* pelvis, *LL* lower lumbar segment, *UL* upper lumbar segment, *LT* lower thoracic segment, *UT* upper thoracic segment

Comparable results were found when reaching laterally (Fig. 6). The thoracic segments showed a significant increase in ROM with reaching height, distance and object weight in the frontal plane (Table 2). The pelvis showed a significant increase in ROM with reaching distance and object weight in this plane. In the sagittal plane, both lumbar segments and the upper thoracic segment showed a significant increase with reaching height, distance and object weight. In the transverse plane, only the pelvis showed a consistent, significant increase in ROM with reaching distance and object weight, but not for reaching height.

**Table 2:** *p* values for the effects of reaching height, distance and object weight on segment range of motion, when reaching laterally

Pair	Reaching height			Reaching distance		Object weight	
	N-S-0/N-E-0	N-S-500/N-E-500	F-S-0/F-E-0	N-S-0/F-S-0	N-E-0/F-E-0	N-S-0/N-S-500	N-E-0/N-E-500
Segment							
Sagittal plane							
Pelvis	0.221	0.879	0.346	0.061	0.627	0.475	0.248
Lower lumbar	0.058	**0.026**	0.548	< **0.001**	**0.005**	< **0.001**	**0.001**
Upper lumbar	**0.002**	**0.002**	0.189	**0.002**	**0.034**	**0.030**	**0.003**
Lower thoracic	0.122	**0.002**	0.083	0.093	0.158	0.069	**0.003**
Upper thoracic	**0.045**	0.946	**0.005**	< **0.001**	**0.001**	**0.006**	0.619
Frontal plane							
Pelvis	0.443	0.761	**0.007**	< **0.001**	< **0.001**	< **0.001**	< **0.001**
Lower lumbar	0.054	**0.013**	0.527	< **0.001**	**0.006**	0.668	0.121
Upper lumbar	0.054	0.093	< **0.001**	**0.007**	0.932	0.074	0.757
Lower thoracic	< **0.001**	< **0.001**	< **0.001**	**0.003**	**0.032**	**0.001**	**0.003**
Upper thoracic	**0.017**	**0.026**	**0.004**	< **0.001**	< **0.001**	**0.001**	**0.007**
Transverse plane							
Pelvis	0.201	0.301	**0.001**	**0.032**	< **0.001**	< **0.001**	**0.001**
Lower lumbar	**0.025**	0.855	**0.016**	0.376	0.833	0.732	0.055
Upper lumbar	0.696	0.476	0.648	0.209	0.549	0.288	0.427
Lower thoracic	**0.001**	0.109	0.382	< **0.001**	0.201	**0.001**	**0.006**
Upper thoracic	0.677	0.925	0.459	**0.020**	**0.013**	0.242	0.201

Bold values indicate significant differences between reaching conditions (post hoc Wilcoxon test)

Abbreviations in reaching tasks: *N* near target, *F* far target, *S* shoulder height, *E* eye height, *0* 0 g object weight, *500* 500 g object weight

**Fig. 7:**
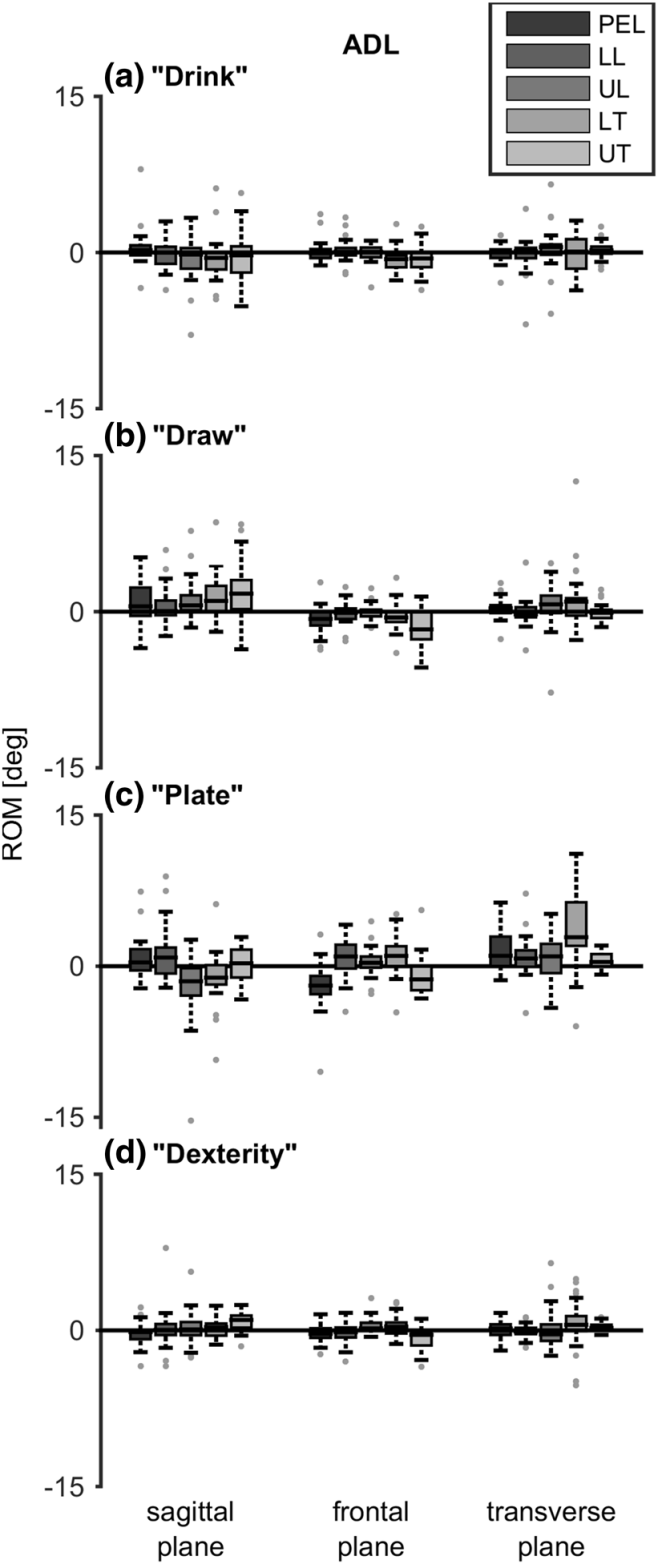
Range of motion (ROM) when performing four activities of daily life. Positive values indicate, respectively, flexion, lateral bending to the right and rotation to the right. Boxes represent 25th, 50th and 75th percentile, whiskers minimum and maximum of non-outlier values, and dots indicate outliers (greater than 1.5 times the interquartile range). *p < 0.05. *PEL* pelvis, *LL* lower lumbar segment, *UL* upper lumbar segment, *LT* lower thoracic segment, *UT* upper thoracic segment

Trunk movement could be seen in all planes when performing daily activities (Fig. 7), even though the activities were within arm length distance. However, the median ROM was often close to zero. Of all the performed tasks, drawing seemed to be the only task where the more cranial trunk segments showed a flexion movement.

Statistical analyses for differences in ROM between segments when performing reaching or ADL tasks, were not performed because of the high variance due to the fact that no specific instructions were given how to perform the tasks. This made it questionable what a significant difference would indicate. Nevertheless, note that the distribution of ROM over trunk segments in all reaching and daily tasks seems quite comparable with the contribution found when performing the maximum trunk movement tasks. The thoracic segments were mostly involved in lateral bending, the lower thoracic segment was mostly involved in axial rotation and the distribution in ROM in the sagittal plane was approximately equal between all trunk segments. However, the movement direction of the segments differed in the sagittal plane; the more caudal segments showed flexion, while more cranial segments showed extension.

### Maturation

**Fig. 8:**
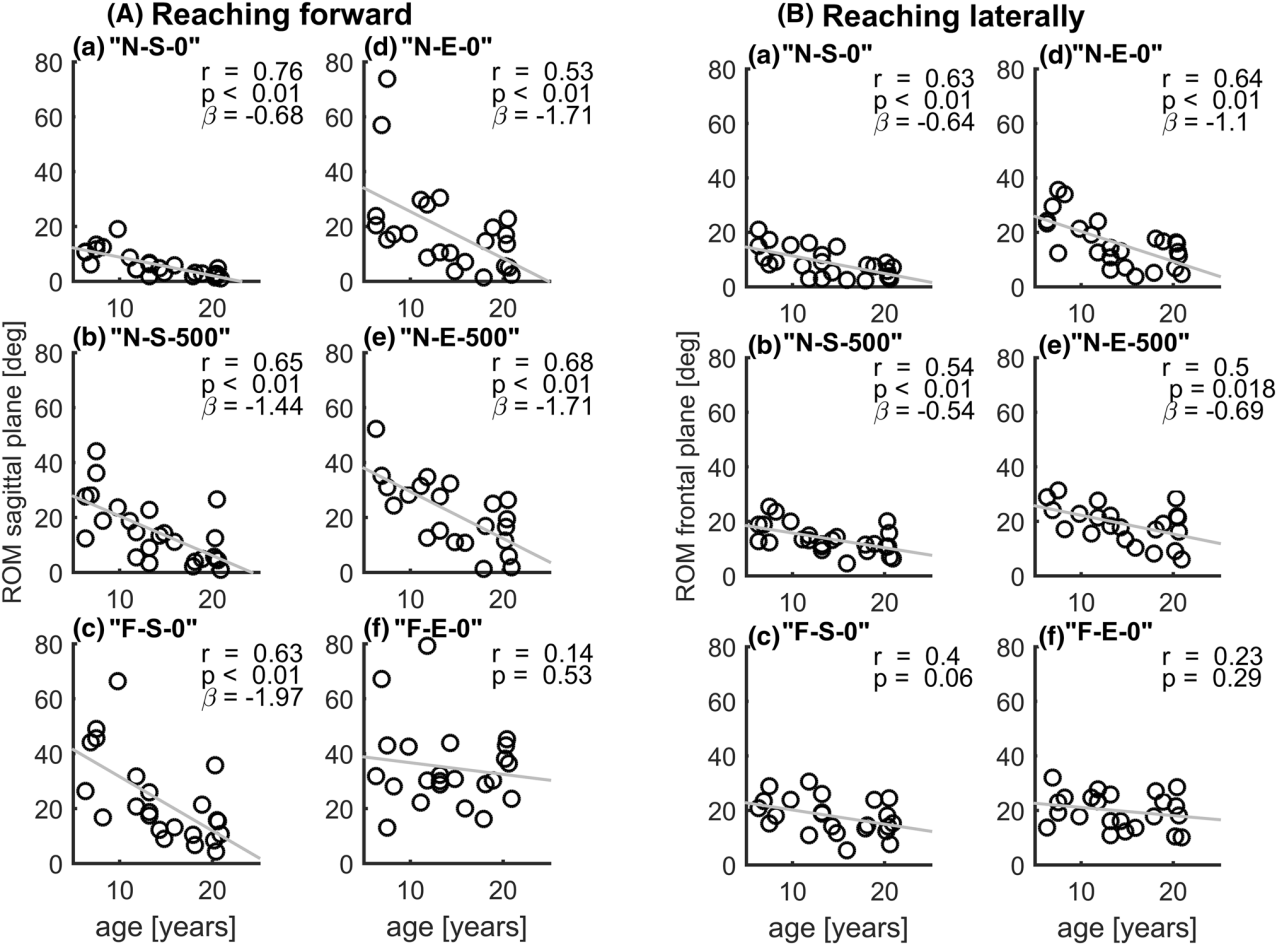
Linear regression between subject age and total trunk range of motion (ROM) in the sagittal plane when reaching forward (left) and in the frontal plane when reaching laterally (right) at different reaching heights, distances and object weights

Figure 8 shows the correlation between age and total trunk movement when reaching forward and laterally. Significant, moderate to strong correlations were found in 10 out of 12 reaching tasks, where younger children used more trunk movement compared to older children. However, a relatively high variability could be seen in the younger children and this variability was higher in reaching forward compared to reaching laterally. The slopes of the regression lines indicated a decrease of trunk ROM of maximal − 1.94°/year for the “F-S-0” task forward and minimal of − 0.54°/year for the “N-S-500” task laterally.

### Head movement strategies

**Fig. 9:**
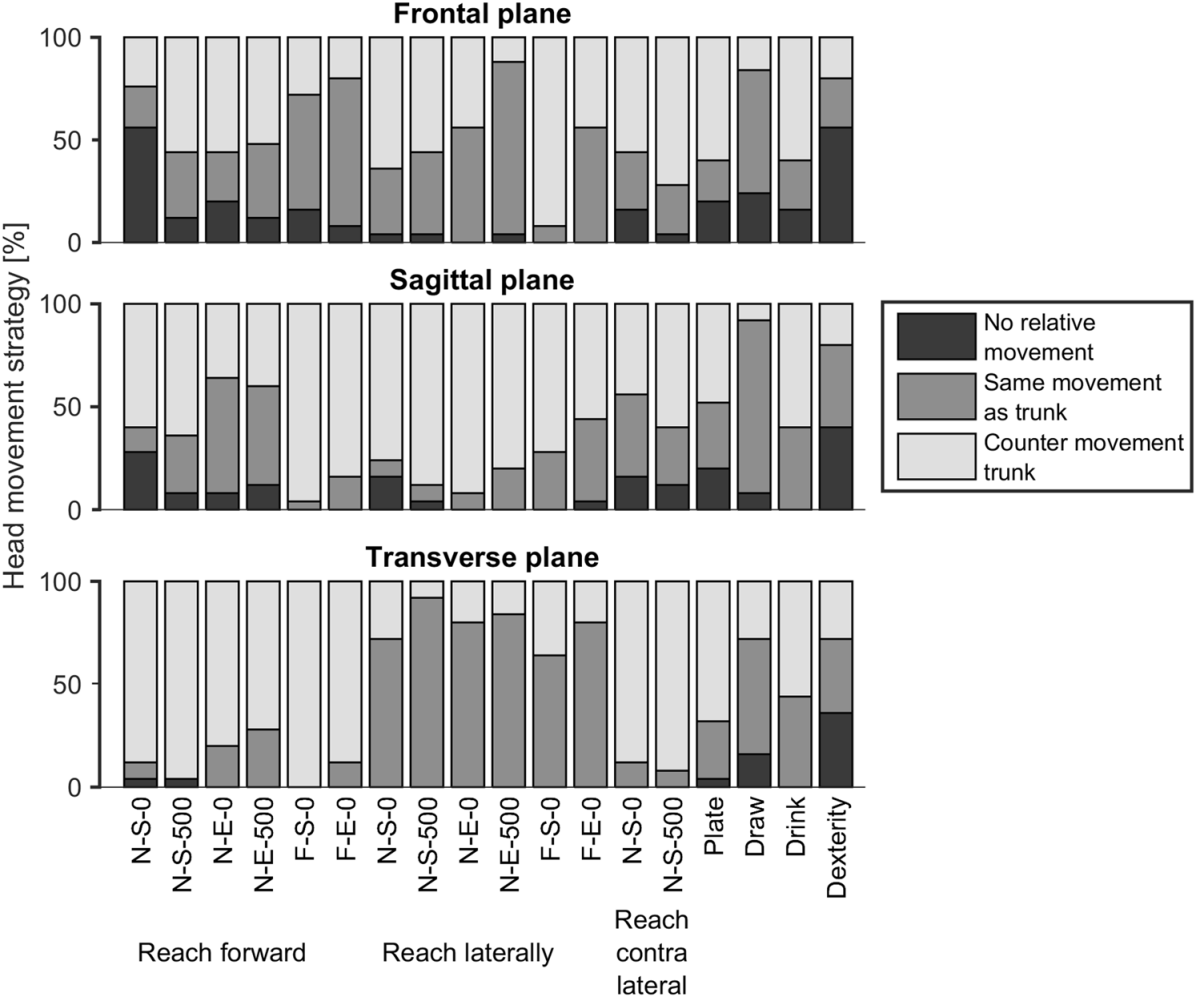
Head movement strategies used by the subjects as percentages of the total group when performing daily tasks. The bars indicate no relative movement between head and trunk, relative head movement in the same direction as the trunk movement and relative head movement in opposite direction of the trunk movement

Different head movement strategies were found in the daily activities (Fig. 9). There was no missing data. Two times the maximum standard deviation of quiet sitting was equal to 2.24° (frontal plane), 2.80° (sagittal plane), 2.02° (transverse plane), and was used as range where the head movement was categorized as none.

In almost all tasks, a variety in head movement strategies was used by the participants. Most consistency could be seen in the transverse plane. Axial rotation movement of the head was in opposite direction to the axial rotation of the trunk when reaching forward and contra-lateral (on average across tasks, 88% of the participants), while the rotation was in the same direction when reaching laterally (on average across tasks, 79% of the participants). In the sagittal plane, more than 50% of the participants moved their head in opposite direction to the trunk. However, both for reaching forward and contra-laterally a substantial part of the participants moved their head in the same direction as the trunk (on average of tasks, 30% of the participants). In the frontal plane, 22% of all participants did not move their head relative to the trunk when reaching forward and contra-laterally and when performing daily tasks. This was higher compared to the other movement planes.

For the four daily tasks, the head movement strategy varied. For the dexterity task, more than 36% of the participants did not move their head relative to the trunk in all planes, and when drawing more than half of the participants moved the head in the same direction as the trunk movement in all planes.

### Movement onset

**Fig. 10:**
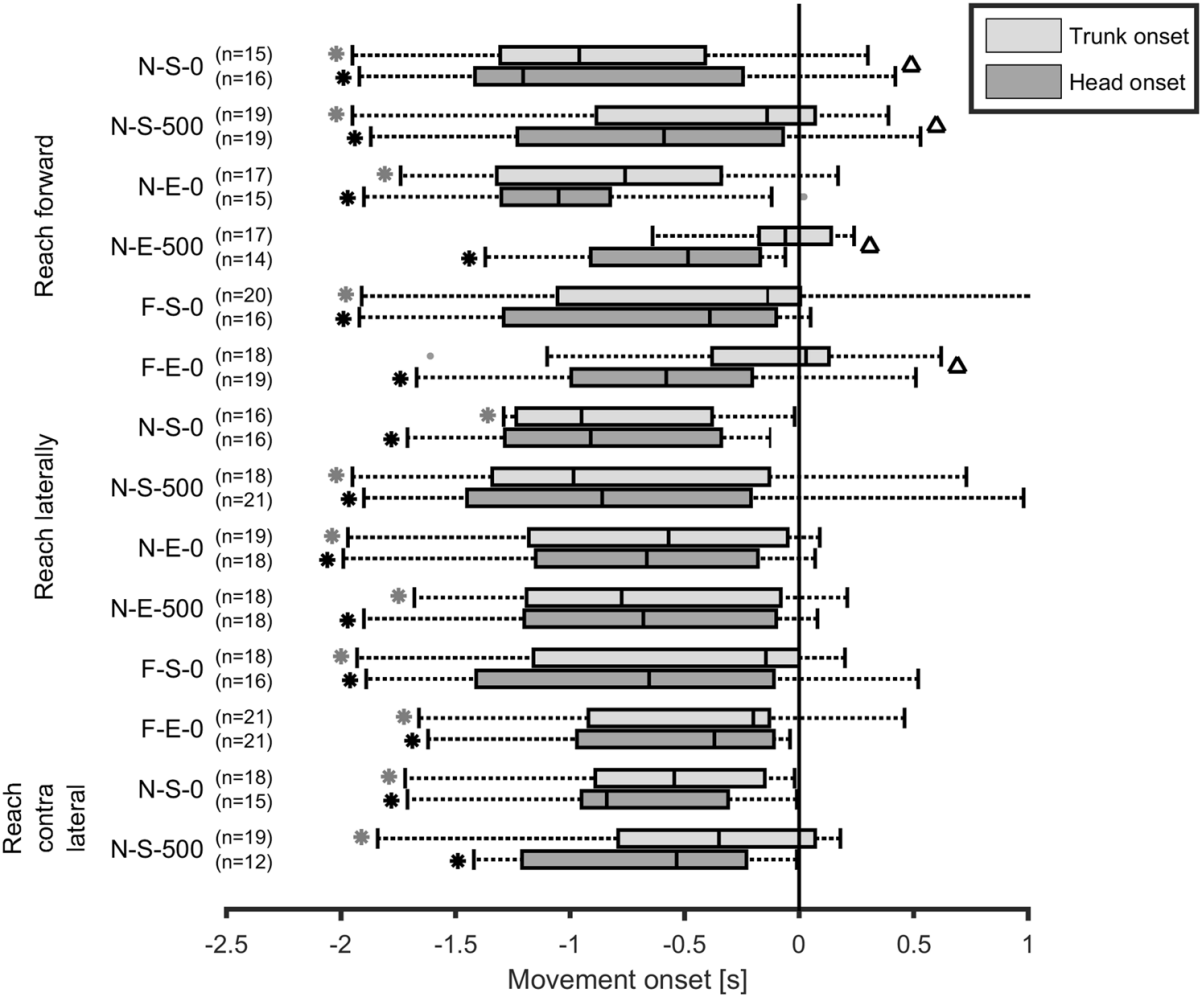
Trunk and head movement onset relative to the arm movement onset for all reaching tasks. Boxes represent 25th, 50th and 75th percentile, whiskers minimum and maximum of non-outlier values, and the dots indicate two outliers above 1.5 times the interquartile range. *A significant difference (*p* < 0.05) for the trunk or head with respect to zero (e.g., arm movement onset) and the ^∆ ^a significant difference (*p* < 0.05) between trunk and head onset

When trunk and head onset were equal to the start of the recording, data were excluded from analysis. It could not be guaranteed that these movements were related to the performed task. The number of included subjects is shown in Fig. 10.

Compared to the arm movement onset, the head movement onset was significantly earlier in all reaching tasks, and the trunk movement onset was significantly earlier in most reaching tasks (Fig. 10). In some tasks when reaching forward, the head onset was also significantly earlier than the trunk onset, resulting in a ‘head–trunk–arm’ movement sequence. However, the interquartile ranges were large and also passed the arm movement onset line, indicating that the movement onset for head and trunk was not prior to the arm movement for every subject.

## Discussion

The results of this study give insight in the interaction between arm, trunk, head and pelvis movements when reaching and performing daily tasks, and in the contribution of different trunk segments to the task in children and young adults.

When performing maximum trunk movement tasks, contributions of individual trunk segments varied with movement direction. In flexion, the contribution was roughly equal among all segments, but in lateral bending the thoracic segments contributed more compared to the lumbar segments, and in trunk axial rotation the lower thoracic segment contributed most. This is in agreement with the study of Preuss and Popovic (2010) for axial rotation, where subjects performed target-directed trunk movements. Their results contradict our results in the other two planes. They found the highest contribution in both flexion–extension and lateral bending from the most caudal segments. These differences are likely due to differences in task instructions. Subjects moved their head along with the trunk in our study, whereas they had to touch a reference with their head in the study of Preuss and Popovic (2010). The pelvis also contributed greatly in all movement directions in our maximum trunk movement tasks, indicating that it has a great influence on the maximum trunk movement.

In accordance with a previous study (Choi and Mark 2004), trunk movement increases with reaching distance and object weight when reaching forward. In addition, we found that this also applies for reaching laterally and for different reaching heights. Moreover, it applies to most trunk segments and the pelvis in the sagittal plane and for the thoracic segments in the frontal plane. It is noticeable that despite the large standard deviations in ROM, subjects adapt similarly to differences in reaching conditions in terms of trunk movement. The trunk segments that showed a significant increased ROM with reaching height, distance and object weight, correspond to the segments contributing the most in the maximum trunk movement tasks: in the frontal plane the thoracic segments and in the sagittal plane all trunk segments, with an exception of the lower thoracic segment when reaching laterally. In the transverse plane, there was no consistent, significant increase in trunk movement between all segments and reaching conditions. This could be explained by the fact that too much trunk rotation will cause an overshoot in arm alignment with the target. Although there was no consistent, significant difference found in axial rotation between the different reaching conditions, axial rotation of the lower thoracic segment was present in each reaching task and therefore seems to be necessary. Again, this is consistent with the finding that the lower thoracic segment contributed the most in the maximum trunk rotation task.

When performing the reaching and daily tasks, anterior tilt of the pelvis and flexion in the lower lumbar segment was seen, while extension was seen in the thoracic segments, indicating that subjects prefer to erect their trunk (decrease thoracic kyphosis and lumbar lordosis) when performing arm tasks. This is in line with suggestions that an erect sitting posture has benefits compared to a slumped posture when performing arm tasks, as it elongates the spine so less arm elevation is needed, and consequently less arm muscle strength, and it ensures a larger shoulder range of motion (Kanlayanaphotporn 2014). Also, the maximum range of axial rotation of the trunk itself increases with a more erect sitting posture (Edmondston et al. 2007).

Strong correlations were found between total trunk ROM and age when reaching forward and laterally. Younger children used more trunk movement compared to older children and the variability was higher, indicating maturation of coordination between trunk and arm movements. The strongest correlations were found when reaching near, at shoulder height and without weight, but the effect (in degrees per year) was the least. This maturation effect with age is in line with findings of Schneiberg et al. (2002) and Sveistrup et al. (2008), and should be taken into account when evaluating children with NMD. Age-matched comparison is very important to distinguish between natural and pathologic trunk movements.

Interactions between trunk and head could already be seen when performing maximal head movements, where the upper thoracic segment contributed quite substantially to (mainly) the maximum neck flexion and extension movement, in agreement with Tsang et al. (2013). When performing daily tasks, the chosen strategy for head movement relative to the trunk, likely depends on maintaining, or achieving, gaze on the target (Land 2006). This could be seen in the transverse plane, where axial rotation of the head was used in the opposite direction to the trunk when reaching forward and contra-laterally, compared to movement in the same direction when reaching laterally. In the sagittal plane, the strategy to move the head in opposite direction of the trunk was most frequently present. However, also a quite substantial percentage of participants did move the head in the same direction as the trunk in several tasks. Variations in strategy might be explained by the relatively small trunk movements, which do not strongly influence the gaze on the object when the head would not move relative to the trunk at all.

Movement onset of the head and the trunk generally seemed to be earlier than the movement onset of the arm when reaching. Only in a few forward reaching tasks, there was also a significant difference between head and trunk onset, resulting in the onset sequence “head–trunk–arm”. These findings correspond to previous literature (Land 2006; Verheyden et al. 2011), however, the variability of movement onset was very large in our study. This could be caused by the chosen method in this study; we did not instruct participants to sit as quietly as possible before the start. Especially for the younger, more energetic subjects, it was difficult to sit quietly. We did ask the participants to look ahead at the beginning of each trial, but especially younger children did not always comply. We tried to eliminate these movements unrelated to the task performed, by excluding the trials in which subjects already moved their head or trunk at the start of the recording before performing the task.

The following considerations should be taken into account when developing new trunk or head supportive devices. Allowing movement between the pelvis and lower lumbar segment is of importance for all movement directions. Based on the relative motions of the lower thoracic segment, allowing movement between the lower thoracic and upper lumbar segments is important for both lateral bending and axial rotation. In addition, since movement of the upper thoracic relative to the lower thoracic segment is quite substantial when bending laterally and when flexing forward, some movement should also be allowed between upper and lower thoracic segments. Although the four trunk segments taken into account in this study still represent a simplification of reality, this analysis provides insight in the minimal degrees of freedom that should be allowed for performance of daily tasks. For a head supportive device, it is important to realize that the head is often moving in the opposite direction of the trunk. Therefore, supportive devices should allow for head rotations independent of the trunk movement. When developing actuated trunk and head supportive devices, they cannot be controlled based on the arm movement when timing of movement is seen as an important factor, since the trunk and head generally started to move prior to the arm movement.

Several other limitations of this study warrant some discussion. First, reaching distance and height were set based on the sitting posture of the subject at the given moment, while small changes in posture may influence reaching distance and height. This may have resulted in variance between trials within and between subjects. However, we were interested in self-selected movements of the trunk and hence chose not to standardize initial sitting posture. Second, although the age of the subjects was uniformly distributed over the whole age range, we had only a few participants for each age. Especially because the variability was larger in the younger children, a larger group size would have allowed for a more sensitive analysis of age effects. Third, surface markers were used to identify movement of the segments. Soft tissue movement can result in artifacts in the movement estimation and is a well-known disadvantage of this measurement technique. Especially the soft tissue movement artifacts of the trunk can be quite substantial (Zemp et al. 2014). However, this influence should be minor when evaluating the range of motion instead of absolute angles according to Zemp et al. (2014). Finally, results of the lumbar segment movement in the younger children should be interpreted with caution, because the markers were placed at a small distance from each other and therefore small artifacts can result in substantial errors.

In conclusion, the contribution of individual trunk segments to the ROM varied with the movement plane with specific task aspects such as distance, height and weight handled. Range of trunk movement decreased with age when performing reaching tasks and this should be kept in mind when evaluating the interaction between trunk and upper extremity movements in children. Increased reaching distance, height and object weight all resulted in increased trunk movement in reaching forward and laterally. Generally, the head moved in opposite direction to the trunk (except in the transverse plane when reach laterally), but the head movement strategy was highly variable in the frontal plane and was also dependent on the task performed. Head and trunk movement onsets were generally earlier than arm movement onset when reaching. Only in a few tasks head movement onset was significantly different from trunk movement onset.
